# Diversification of Two Lineages of Symbiotic *Photobacterium*

**DOI:** 10.1371/journal.pone.0082917

**Published:** 2013-12-13

**Authors:** Henryk Urbanczyk, Yoshiko Urbanczyk, Tetsuya Hayashi, Yoshitoshi Ogura

**Affiliations:** 1 Interdisciplinary Research Organization, University of Miyazaki, Miyazaki, Japan; 2 Division of Bioenvironmental Science, Frontier Science Research Center, University of Miyazaki, Miyazaki, Japan; 3 Division of Microbiology, Department of Infectious Diseases, Faculty of Medicine, University of Miyazaki, Miyazaki, Japan; Radboud University Medical Centre, NCMLS, Netherlands

## Abstract

Understanding of processes driving bacterial speciation requires examination of closely related, recently diversified lineages. To gain an insight into diversification of bacteria, we conducted comparative genomic analysis of two lineages of bioluminescent symbionts, *Photobacterium leiognathi* and ‘*P. mandapamensis*’. The two lineages are evolutionary and ecologically closely related. Based on the methods used in bacterial taxonomy for classification of new species (DNA-DNA hybridization and ANI), genetic relatedness of the two lineages is at a cut-off point for species delineation. In this study, we obtained the whole genome sequence of a representative *P. leiognathi* strain *lrivu.*4.1, and compared it to the whole genome sequence of ‘*P. mandapamensis*’ *svers.*1.1. Results of the comparative genomic analysis suggest that *P. leiognathi* has a more plastic genome and acquired genes horizontally more frequently than ‘*P. mandapamensis*’. We predict that different rates of recombination and gene acquisition contributed to diversification of the two lineages. Analysis of lineage-specific sequences in 25 strains of *P. leiognathi* and ‘*P. mandapamensis*’ found no evidence that bioluminescent symbioses with specific host animals have played a role in diversification of the two lineages.

## Introduction

*Photobacterium* (*Vibrionaceae*, Gammaproteobacteria) are ubiquitous marine bacteria [1]–[2]. Research of *Photobacterium* can be traced back to the 19th century, when studies of luminous bacteria by Martinus W. Beijerinck led to description of the genus [3]. Later studies found that some *Photobacterium* can form bioluminescent symbioses with marine fish and squids [2], [4]–[6]. In these associations, the animal forms a gland-like tissue called light organ, which is colonized by symbiotic bacteria. These symbiotic bacteria live extracellulary inside the light organ in dense communities and produce luminescence that is diffused from the host body [2], [7]–[12]. *Photobacterium* associations with the host animals are not obligate, and the symbionts can survive and reproduce outside of the host.

Early systematics of *Photobacterium* relied on phenotypic traits and DNA-DNA hybridization to distinguish between species in the genus [2]. Modern taxonomic studies of *Photobacterium* rely on multilocus sequence analyses, which make use of sequences of multiple genes for delineation of species [2]. Multilocus analyses using sequences of rRNA genes or sequences of genes coding for housekeeping proteins allowed resolving evolutionary relationship between most species in the genus [2]. However, multilocus sequence analyses using sequences of housekeeping genes could not resolve the controversy regarding evolutionary relationship and taxonomic classification of two lineages of bioluminescent symbionts, *Photobacterium leiognathi* and ‘*P. mandapamensis*’. Originally described by Boisvert et al. [13] and Hendrie et al. [14] as separate species, the two lineages were synonymized under the name *P. leiognathi* by Reichelt and Baumann [15] after throughout phenotypic and chemotaxonomic analyses. Ast and Dunlap [16] found that *P. leiognathi* could be separated into two lineages by phylogenetic analyses of luminescence genes (*luxCDAB*(*F*)*E* [2]), but analysis of sequences of 16S rRNA gene or a housekeeping gene *gyrB* could not resolve distinct clades. Based on these results Ast and Dunlap [16] proposed dividing *P. leiognathi* into two subspecies, *P. leiognathi* subsp. *leiognathi* and *P. leiognathi* subsp. *mandapamensis*. Wada et al. [17] also found that *P. leiognathi* could be divided into two lineages using phylogenetic analyses of sequences of luminescence genes. Each of the two lineages included the type strains proposed for *P. leiognathi* and ‘*P. mandapamensis*’ (ATCC 25521^T^ and ATCC 27561^T^, respectively) in original descriptions of the species [13]–[14]. Similar to Ast and Dunlap [16], Wada et al. [17] also could not resolve the two lineages by analysis of sequences of 16S rRNA gene. Further studies of the two lineages led Kaeding et al. [6] to propose a revival of ‘*P. mandapamensis*’ as a species distinct from *P. leiognathi*. The proposal to classify *P. leiognathi* and ‘*P. mandapamensis*’ as distinct species was supported by phylogenetic analyses of nucleotide sequences of luminescence genes as well as ecological studies which have shown that strains from the two lineages have mostly different symbiotic host species, and rarely share the same host fish [6]. However, the use of phylogenetic analyses of sequences of luminescence genes to distinguish between *P. leiognathi* and ‘*P. mandapamensis*’ came into question after discovery of horizontal transfer of luminescence genes [18]–[19]. Evidence of horizontal transfer of luminescence genes from *P. leiognathi* to some strains of ‘*P. mandapamensis*’ [19] challenged the appropriateness of separating the two lineages based on phylogenies of luminescence genes, since the evolutionary relationships of luminescence genes could be different from the organismal relationships.

Frequent taxonomic nomenclature changes and difficulties in distinguishing strains from both lineages reflect close evolutionary relatedness of *P. leiognathi* and ‘*P. mandapamensis*’. Despite difficulties in the taxonomic classification, systematic studies of *P. leiognathi* and ‘*P. mandapamensis*’ have shown that these bioluminescent symbionts form two closely related, discrete lineages. Studies of closely related lineages such as *P. leiognathi* and ‘*P. mandapamensis*’ can offer a unique insight into bacterial diversification, and improve our understanding of evolution of bacterial species. To begin investigating diversification of *P. leiognathi* and ‘*P. mandapamensis*’, we initiated genomic comparison studies of representative strains from both lineages. Comparative analysis of whole genome sequences of strains from both lineages revealed differences in the plasticity of *P. leiognathi* and ‘*P. mandapamensis*’ genomes, as well as differences in the frequency of recombination and horizontal acquisition of genes between both lineages. We also found novel markers for phylogenetic analyses, which allow delineating *P. leiognathi* and ‘*P. mandapamensis*’ strains. We also looked for evidence of ecological diversification, i.e. whether diversification of *P. leiognathi* and ‘*P. mandapamensis*’ could be explained by gain or loss of genes necessary to form symbiotic associations with a specific animal host.

## Materials and Methods

### Ethic statement

An ethic statement is not required for this study according to the ethical guidelines for animal studies of the University of Miyazaki. Fish were kindly provided by our colleague in the Faculty of Agriculture, Yukio Iwatsuki. Handling and sacrifice of the fish followed procedures required by University of Miyazaki; no specific approval or permits were required, and the study did not involved endangered or protected species. Fish were caught using trawls or trap nets, and sacrificed in an ice water bath, to preserve both the light organ and the bacteria.

### Bacterial strains and culture conditions

Strains with designations beginning with 170910F and 220710F were isolated from light organs of fish collected for research purpose only, during a research trip by researchers at the University of Miyazaki. Fish were collected from trawls or trap nets off the coast of the Kyushu Island (Japan) (see Table 1 for the list of fish species). Fish were sacrificed for research purposes only, by researchers at the University of Miyazaki; fish scarification was done by immersion in an ice water bath, in order to preserve microbial samples. Other methods of sacrificing fish specimens, including decapitation and use of chemicals, could potentially endangered light organ symbionts or introduce spoilage bacteria into the light organ tissue. The fish were kept on ice until bacterial analysis. Light organ dissection followed procedure described previously [1]–[2]. Other bacterial strains used in this study were previously collected from light organs of bacterially luminous fishes, from skin of marine fishes, or isolated directly from seawater [2], [6], [16], [18] (see Table 1 for information about ecological source of the strains). Bacteria were grown in LSW-70 broth [1] which contained per liter 10 g tryptone, 5 g yeast extract, 350 ml double-strength artificial seawater and 650 ml of deionized water. For solid medium 15 g of agar per liter were added. Bacteria were grown at a room temperature with slow aeration.

**Table 1:** *P. leiognathi* and ‘*P. mandapamensis*’ strains used in this study.

Species	Strain name	Exopolysaccharide biosynthesis genes^a^						Ecological source^b^
		(a)	(b)	(c)	(d)	(e)	(f)	
*P. leiognathi*	*lrivu*.4.1	+	+	−	−	−	−	LO, *Equulites rivulatus*
‘*P. mandapamensis*’^c^	*lrivu*.3.1	−	−	−	−	+	+	LO, *Equulites rivulatus*
‘*P. mandapamensis*’^c^	220710F8A	−	−	−	−	+	+	LO, *Equulites rivulatus*
‘*P. mandapamensis*’^c^	220710F9A	+	+	−	−	+	+	LO, *Equulites rivulatus*
‘*P. mandapamensis*’^c^	220710F10A	−	−	−	−	+	+	LO, *Equulites rivulatus*
‘*P. mandapamensis*’^c^	220710F10B	−	−	+	+	+	+	LO, *Equulites rivulatus*
*P. leiognathi*	*lnuch*.13.1	−	−	−	−	−	−	LO, *Nuchequula nuchalis*
*P. leiognathi*	*lnuch*.21.1	−	−	−	−	−	−	LO, *Nuchequula nuchalis*
*P. leiognathi*	170910FA1	−	−	+	+	−	−	LO, *Nuchequula nuchalis*
*P. leiognathi*	170910FC1	−	−	+	+	−	−	LO, *Nuchequula nuchalis*
‘*P. mandapamensis*’^c^	170910FB1	−	−	+	+	+	+	LO, *Nuchequula nuchalis*
*P. leiognathi*	220710F2A	−	−	−	−	−	−	LO, *Secutor indicius*
*P. leiognathi*	220710F4A	−	−	−	−	−	−	LO, *Secutor indicius*
*P. leiognathi*	220710F5A	−	−	−	−	−	−	LO, *Secutor indicius*
‘*P. mandapamensis*’^c^	220710F3A	−	−	+	+	+	+	LO, *Secutor indicius*
‘*P. mandapamensis*’	*svers*.1.1	−	−	+	+	+	+	LO, *Siphamia versicolor*
‘*P. mandapamensis*’	*svers*.9.9	−	−	+	−	+	+	LO, *Siphamia versicolor*
‘*P. mandapamensis*’	*ajapo*.3.1	−	−	−	−	+	+	LO, *Acropoma japonicum*
‘*P. mandapamensis*’	*ajapo*.4.20	+	+	−	−	+	−	LO, *Acropoma japonicum*
*P. leiognathi*	ATCC 25521^T^	−	−	+	+	−	−	LO, *Leiognathus splendens*
‘*P. mandapamensis*’^c^	*ppana*.3.1	−	−	−	−	+	+	LO, *Photopectoralis panayensis*
*P. leiognathi*	*lelon*.2.1	+	+	−	−	−	−	LO, *Photopectoralis elongatus*
‘*P. mandapamensis*’	PL−721	−	−	+	+	+	+	Skin isolate, *Coccorella* sp.
‘*P. mandapamensis*’	NCCB 80036	−	−	−	−	+	+	Seawater isolate, Netherlands
‘*P. mandapamensis*’	ATCC 27561^T^	−	−	+	+	+	+	Seawater isolate, Indonesia

+) or absence (−) of exopolysaccharide biosynthesis genes in the analyzed strains. Letters correspond to amplification scheme shown in the Figure 2.^a^ Presence (

^b^ LO: light organ of a fish, name of the fish host is provided.

‘*P. mandapamensis*’ strains previously identified as *P. leiognathi* based on analysis of luminescence genes^c^

### Genome sequencing

A draft genome sequence of *P. leiognathi lrivu*.4.1 was obtained using the Roche 454 GS FLX titanium platform. We obtained 237,621 single-end reads (average read length 427 bp) and 450,573 paired-end reads (8 kb fragments), with approximately 33-fold coverage. The sequence was initially assembled using GS Assembler into 275 contigs of over 500 bp (N50 contig size is 52,884 bp). Gap closing on the scaffold that included a complete sequence of a large plasmid of 159 kb was performed using primer walking, closing all 10 gaps in the scaffold sequence. Identification of protein-coding sequences (CDSs) was carried out using Microbial Annotation Pipeline (MiGAP) [20] with additional information provided by Manatee (IGS Annotation Service [http://manatee.sourceforge.net]). The sequence was deposited in DDBJ under accession numbers BANQ01000001 to BANQ01000184. The draft genome sequence of ‘*P. mandapamensis*’ *svers.*1.1 was previously determined by our group (accession number BACE00000000.1) [21]. ‘*P. mandapamensis*’ *svers*.1.1 genes have the locus tag PMSV and *P. leiognathi lrivu*.4.1 genes have the locus tag PLEI.

### Comparative genomics

Lists of orthologs shared by *P. leiognathi lrivu*.4.1 and ‘*P. mandapamensis*’ *svers*.1.1 (accession number BACE00000000.1) [21], *P. angustum* S14 (accession number AAOJ00000000.1) [22] and *P. angustum* SKA34 (accession number AAOU00000000) [23], *P. profundum* SS9 (accession number PRJNA62923) [24] and *P. profundum* 3TCK (accession number AAPH00000000) were identified for each pair of strain by reciprocal BLASTP searches using an e-value cut-off of 1e-5, with no more than 50% sequence divergence over the entire alignment of the sequence. A list of orthologs shared by ‘*P. mandapamensis*’ *svers*.1.1 and *P. leiognathi lrivu*.4.1 was used to identify markers for phylogenetic analysis of *P. leiognathi* and ‘*P. mandapamensis*’. In order to find sequences unique to ‘*P. mandapamensis*’ *svers.*1.1 (i.e. absent in *P. angustum* S14 and *P. leiognathi lrivu*.4.1), the lists of orthologs shared by ‘*P. mandapamensis*’ *svers*.1.1 and *P. angustum* S14, or by ‘*P. mandapamensis*’ *svers*.1.1 and *P. leiognathi lrivu*.4.1 were removed from a list of all ‘*P. mandapamensis*’ *svers.*1.1 CDSs to produce a list of sequences unique to ‘*P. mandapamensis*’ *svers*.1.1. Similarly, genes unique to *P. leiognathi lrivu*.4.1 (i.e. absent in *P. angustum* S14 or ‘*P. mandapamensis*’ *svers.*1.1) were found by comparing sets of orthologous genes shared by *P. leiognathi lrivu*.4.1 and *P. angustum* S14, and by comparing sets of orthologous genes shared by *P. leiognathi lrivu*.4.1 and ‘*P. mandapamensis*’ *svers.*1.1 to a list of all *P. leiognathi lrivu*.4.1 CDSs. COG assignment of genes was done using WebMGA web server (http://weizhong-lab.ucsd.edu/metagenomic-analysis/server/cog/) [25]. Transposases present in both genomes were identified after search of the annotations by keywords and by using the IS Finder database (http://www-is.biotoul.fr/).

### ANI

Average nucleotide identity (ANI) was calculated by the JSpecies program [26] version 1.2.1 using ANIb with default settings, i.e. BLASTN options: -X 150 -q -1 -F F -e 1-15 -a 2; fragments length: 1020; fragments alignment length: 70% or longer; and fragments identity: 30% or higher. The draft genome sequence of *P. leiognathi lrivu*.4.1 includes significant number of large gaps, which hindered ANI calculations using JSpecies. Specifically, JSpecies could not finish calculating ANI based on BLASTN results that included large number of query sequences consisting of 1020 Ns. Therefore, after chopping *P. leiognathi lrivu.*4.1 sequence fragments that included 1020 Ns were removed from the calculations. Other genome sequences were not altered for calculations of ANI.

### DDH

DNA-DNA hybridization experiments were performed at the TechnoSuruga Company (Japan), as described by [27]. Hybridization temperature was 41°C, except for the hybridization between strains ATCC 25521^T^ and ATCC 27561^T^, which was performed at 42°C. DNA-DNA relatedness data were provided as mean values of four measurements.

### DNA amplification and sequencing

Bacterial DNA was isolated using a DNeasy Blood & Tissue kit (Qiagen). PCR amplification was performed using GoTaq Green Master Mix (Promega) using the manufacturer's protocol. Sequences of all primers used in this study can be found in the Table S1. Annealing temperatures used were 48°C or 49°C. PCR amplified DNA was sequenced using an ABI Prism 3100 Genetic Analyzer (Applied Biosystems) with an ABI Prism Big Dye Terminator Cycle Sequencing Ready Reaction Kit (Applied Biosystems). Accession numbers for sequences used in the study are listed in the Table S2.

### Phylogenetic analyses

Sequences were manually aligned in the MacClade 4.08 software [28]. Accession numbers for sequences used in the phylogenetic analyses are listed in the Table S1. The alignments were used for phylogenetic analyses with parsimony criterion using TNT [29]. The searches were done using 100 replicates of new search technology (using xmult = level10 replic 100; command), jackknife resampling values were calculated after 10,000 replicates (using resample = jak probability 34 frequency replications 10000; command). Phylogenetic trees were visualized in the FigTree v1.3.1 software (http://tree.bio.ed.ac.uk/software/figtree/).

## Results

### Strains and general genome overview

Two strains analyzed in this study, *P. leiognathi lrivu*.4.1 and ‘*P. mandapamensis*’ *svers*.1.1, were isolated from symbioses with bioluminescent fish. *P. leiognathi lrivu*.4.1 was isolated form a light organ of bacterially bioluminescent leiognathid fish *Photoplagios rivulatus* [18]. ‘*P. mandapamensis*’ *svers*.1.1 is the bioluminescent symbiont of the cardinal fish *Siphamia versicolor* [21]. The two strains are representative for *P. leiognathi* and ‘*P. mandapamensis*’, respectively.

Whole genome sequences of ‘*P. mandapamensis*’ *svers*.1.1 and *P. leiognathi lrivu*.4.1 consist of 38 and 275 contigs, respectively, assembled into 12 and 20 scaffolds, respectively. Estimated length of the whole genome sequences of ‘*P. mandapamensis*’ *svers*.1.1 and *P. leiognathi lrivu*.4.1 are 4.56 Mb and 5.27 Mb, respectively. Both genomes consist of two circular chromosomes (as confirmed by a pulsed-field electrophoresis [PFGE] analysis; data not shown), similar to other species in the family *Vibrionaceae*. A large plasmid of 159 kb was found in *P. leiognathi lrivu*.4.1, while no plasmids were detected in ‘*P. mandapamensis*’ *svers*.1.1. *P. leiognathi lrivu*.4.1 appears to have two copies of operon that codes for luminescence genes, with one copy of the operon on the 159 kb plasmid. The G+C content of ‘*P. mandapamensis*’ *svers.*1.1 is 42.25% while that of strain *P. leiognathi lrivu*.4.1 is 40.56%.

The predicted genome sequence of *P. leiognathi lrivu*.4.1 is 14.6% larger than the genome sequence of ‘*P. mandapamensis*’ *svers*.1.1. The difference between genome sizes of the two strains is greater than the differences between genomes of other *Photobacterium* species for which whole genome sequence data is available. Genome sequence of *P. profundum* SS9 is 3.5% larger than the genome sequence of *P. profundum* 3TCK, while the genome sequence of *P. angustum* S14 is only 0.8% larger than the genome of *P. angustum* SKA34.

### Genome comparison of symbiotic *Photobacterium*

The genome of ‘*P. mandapamensis*’ *svers*.1.1 encodes 4,026 CDSs including 346 hypothetical CDSs (8.6% of the total CDSs). The genome of *P. leiognathi* strain *lrivu*.4.1 encodes 4,351 CDSs including 1,188 hypothetical CDSs (27.3% of the total CDSs). Functional analysis of genes present in ‘*P. mandapamensis*’ *svers*.1.1 and *P. leiognathi lrivu*.4.1 based on the COG functional classification revealed that both genomes have similar distribution of CDSs in most COG categories (Table 2). As expected, *P. leiognathi lrivu*.4.1 had higher percentage of CDSs with unknown function that could not be assigned to any COG group. Differences between the two strains were apparent in the replication, recombination and repair class of genes (COG category L), which were significantly more numerous in the *P. leiognathi lrivu*.4.1 genome. Inspection of *P. leiognathi lrivu*.4.1 CDSs in the COG category L revealed proliferation of sequences annotated as transposases. In total, the strain *P. leiognathi lrivu*.4.1 had 77 transposases which were assigned to 11 different IS elements families. Transposases from IS *21*, IS *6* and IS *66* families were the most abundant in *P. leiognathi lrivu.*4.1, while 30 transposases were not assigned to any family. Thirty-three of the transposases were found on the 159 kb plasmid, 44 transposases were located on the chromosomes. ‘*P. mandapamensis*’ *svers*.1.1 genome had only one CDS annotated as a transposase, which was not assigned to any IS family.

**Table 2:** Comparative analysis of *P. leiognathi* and ‘*P. mandapamensis*’ proteins.

COG category	*lrivu*.4.1 total	*svers*.1.1 total	*lrivu*.4.1 specific	*svers*.1.1 specific	Category description
-	12.35%	6.31%	59%	55%	Not in COGs
A	0.02%	0.02%	0%	0%	RNA processing and modification
B	0.02%	0.02%	0%	0%	Chromatin structure and dynamics
C	6.09%	6.43%	1%	1%	Energy production and conversion
D	1.08%	1.12%	1%	1%	Cell cycle control, cell division, chromosome partitioning
E	6.78%	7.53%	0%	1%	Amino acid transport and metabolism
F	2.05%	2.26%	0%	1%	Nucleotide transport and metabolism
G	4.14%	4.67%	1%	4%	Carbohydrate transport and metabolism
H	4.19%	4.50%	2%	1%	Coenzyme transport and metabolism
I	2.41%	2.76%	0%	0%	Lipid transport and metabolism
J	4.46%	4.87%	0%	1%	Translation, ribosomal structure and biogenesis
K	5.93%	6.51%	3%	3%	Transcription
L	5.89%	3.95%	14%	6%	Replication, recombination and repair
M	5.57%	6.06%	3%	5%	Cell wall/membrane/envelope biogenesis
N	3.31%	3.48%	1%	2%	Cell motility
O	3.89%	4.20%	1%	2%	Posttranslational modification, protein turnover, chaperones
P	4.30%	5.17%	1%	1%	Inorganic ion transport and metabolism
Q	1.08%	1.24%	0%	0%	Secondary metabolites
R	8.83%	10.36%	4%	9%	General function prediction only
S	7.34%	8.00%	3%	1%	Function unknown
T	5.57%	6.14%	1%	1%	Signal transduction mechanisms
U	3.17%	3.10%	3%	3%	Intracellular trafficking, secretion, and vesicular transport
V	1.52%	1.32%	3%	1%	Defense mechanisms

Comparative analysis of functional group distribution among *P. leiognathi lrivu*.4.1 and ‘*P. mandapamensis*’ *svers*.1.1 proteins based on COGs classification. The data is provided as a percentage of proteins in one specific functional group in function of the total number of proteins. *lrivu*.4.1 specific and *svers*.1.1 specific columns refer to classification of strain specific sequences, i.e. sequences found only in *lrivu.*4.1 (but not in ‘*P. mandapamensis*’ *svers.*1.1 or *P. angustum* S14), or only in ‘*P. mandapamensis*’ *svers*.1.1 (but not in *P. leiognathi lrivu*.1.1 or *P. angustum* S14).

### Whole-genome relatedness of *P. leiognathi* and ‘*P. mandapamensis*’

Two methods used in bacterial taxonomy, DNA-DNA hybridization (DDH) and the average nucleotide identity (ANI), were used to investigate whole genome-relatedness between both lineages. ANI between ‘*P. mandapamensis*’ *svers.*1.1 and *P. leiognathi lrivu*.4.1 was 96.8% when ‘*P. mandapamensis*’ *svers*.1.1 sequence was used as a query and 96.6% when *P. leiognathi lrivu.*4.1 sequence was used as a query. These ANI values are at the 95–96% cut-off proposed for strains from the same species [30]–[31]. For comparison, ANI between ‘*P. mandapamensis*’ *svers.*1.1 or *P. leiognathi lrivu.*4.1 and a five other *Photobacterium* strains from three species was calculated (see Table S3). Both ‘*P. mandapamensis*’ *svers*.1.1 and *P. leiognathi lrivu*.4.1 have ANI of 82.54% or lower to other *Photobacterium* strains, which is below the proposed 95–96% cut-off. It should be noted that ANI between *P. profundum* SS9 and 3TCK and ANI between *P. angustum* S14 and SKA34 is 93.41% or lower, which indicates that the genetic distance between sequences conserved in *P. leiognathi* and ‘*P. mandapamensis*’ is lower than the genetic distance between sequences conserved in *P. profundum* or *P. angustum*.

The DNA reassociation values between the type strains of *P. leiognathi* and ‘*P. mandapamensis*’ were 80.25% when ATCC 25525^T^ (the type strains of *P. leiognathi*) was used as a probe and 74% when ATCC 27561^T^ (the type strain of ‘*P. mandapamensis*’) was used as a probe. DNA reassociation values between type strains of *P. leiognathi* or ‘*P. mandapamensis*’ and *P. angustum* ATCC 25195^T^ were 36.75% and 41.25%, respectively. Current guidelines for microbial classification recommend that bacterial strains with DNA reassociation values of 70% or higher could be considered members of the same species [32]–[33].

ANI calculated for the whole genome sequences of *P. leiognathi lrivu.*4.1 and ‘*P. mandapamensis*’ *svers.*1.1 and the results of and DDH indicate that *P. leiognathi* and ‘*P. mandapamensis*’ should be classified as members of the same species according to strict bacterial taxonomy rules. However, both analyses demonstrate that the two lineages are at the cut-off of the current bacterial species definition.

In addition to ANI, CDSs of ‘*P. mandapamensis*’ *svers.*1.1 and *P. leiognathi lrivu.*4.1 were compared with each other by a pairwise reciprocal BLASTP analysis (see Materials and Methods for details). The comparison revealed that the two strains share 3,453 CDSs (85.7% of the ‘*P. mandapamensis*’ *svers*.1.1 total CDSs and 79.4% of the *P. leiognathi lrivu*.4.1 total CDSs). Searchers using the same method were performed for pairs of strains from two other *Photobacterium* species, *P. angustum* (strains S14 and SKA34) and *P. profundum* (SS9 and 3TCK). Results showed that strains *P. angustum* S14 and SKA 34 share 3,797 CDSs (83.3% of the S14 total CDSs and 83.14% of the SKA34 total CDSs), while strains *P. profundum* SS9 and 3TCK share 4,007 CDSs (73% of the SS9 total CDSs and 72.21% of the 3TCK total CDSs).

### Systematics of P. leiognathi and ‘P. mandapamensis’

In order to establish if *P. leiognathi* and ‘*P. mandapamensis*‘ can be separated using molecular phylogeny, a search for markers suitable for phylogenetic separation of both lineages was initiated. From the 3,453 CDSs shared between ‘*P. mandapamensis*’ *svers.*1.1 and *P. leiognathi lrivu.*4.1, orthologs with alignable sequences shorter than 250 amino acids were excluded from the analysis since they were considered too short for use as markers. Among the remaining orthologs, 46 orthologs with sequence identity between 78.95% (i.e. lowest nucleotide sequence identity found between ‘*P. mandapamensis*’ *svers*.1.1 and *P. leiognathi lrivu*.4.1 orthologs) and 90% were manually analyzed for usefulness in phylogenetic analysis of the two lineages. Specifically, we excluded orthologs that had no matching *Vibrionaceae* sequences in public databases, since phylogenetic analyses require comparison of *P. leiognathi* and ‘*P. mandapamensis*’ sequences in context to related species.

Also, if an analysis of *Vibrionaceae* strains other than *P. leiognathi* and ‘*P. mandapamensis*’ using sequences of a potential marker predicted evolutionary relationship different from the evolutionary relationship predicted when using housekeeping genes, that ortholog was excluded from further analyses (data not shown). Based on these criteria, 6 orthologs were selected for analysis (see supplementary material). Sequences of the 6 orthologs were amplified and sequenced in 8 strains representative for *P. leiognathi* and ‘*P. mandapamensis*’, and used for phylogenetic analyses (see Figure S1 for the results).

**Figure 1:**
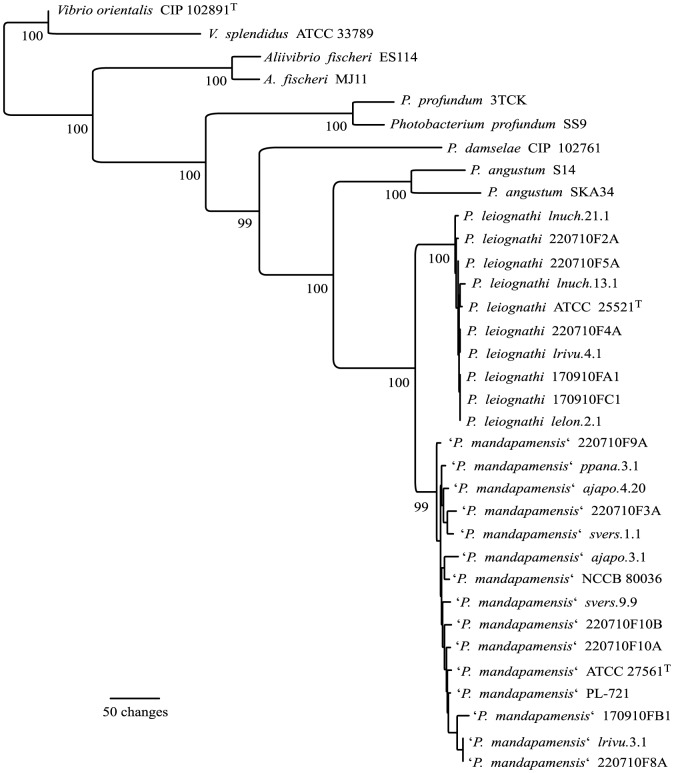
Phylogenetic analysis of *P. leiognathi* and ‘*P. mandapamensis*’. Phylogenetic tree representing 1 of 45 equally parsimonious hypotheses resulting from the analysis of concatenated alignments of nucleotide sequences of PMSV_2285 and PMSV_ 4043 homologs in 25 *P. leiognathi* and ‘*P. mandapamensis*’ strains and related *Vibrionaceae*. Sequence alignment had 1794 characters (including 1020 informative characters). The trees length was equal to 3030. The 45 resulting hypotheses differed in the predicted relationship between strains within *P. leiognathi* and ‘*P. mandapamensis*’ clades. Jackknife resampling values are shown at the nodes, some were omitted for clarity. Analyses of the concatenated PMSV_2285 and PMSV_ 4043 alignment were also carried out using neighbor-joining and maximum-likelihood algorithms; results of these analyses showed the same cladding of *P. leiognathi* and ‘*P. mandapamensis*’ strains (see Figure S2).

Two of the 6 analyzed orthologs were selected as potential markers for use in phylogenetic analyses, a putative HlyD family secretion membrane fusion protein (locus tags PMSV_2285 in ‘*P. mandapamensis*’ *svers*.1.1 and PLEI_3531 in *P. leiognathi lrivu*.4.1) and a secretion pathway protein (locus tags PMSV_4043 in ‘*P. mandapamensis*’ *svers.*1.1 and PLEI_1315 in *P. leiognathi lrivu*.4.1). Phylogenetic analyses using aligned sequences of the two orthologs separated all analyzed *P. leiognathi* and ‘*P. mandapamensis*’ strains into two clades, with *P. leiognathi* strains ATCC 25525^T^ and *lrivu.*4.1 in a different clade than ‘*P. mandapamensis*’ strains ATCC 27561^T^ and *svers.*1.1 (see Figure 1). Sequences of the two genes were amplified in additional fifteen strains from both lineages, for a total of 25 strains. These strains were isolated from symbioses with eight different host species, from non-symbiotic association with fish, or directly form the seawater. Homologs of PMSV_2285 could not be amplified in ‘*P. mandapamensis*’ strain *ajapo.*4.20, despite numerous attempts (data not shown). Sequences of the two genes were concatenated and aligned with those in related *Vibrionaceae* and used for phylogenetic analysis. As shown in Figure 1, the analysis separated 25 analyzed strains into two clades, with strains ATCC 25525^T^ and *P. leiognathi lrivu.*4.1 in a different clade from strains ATCC 27561^T^ and *svers.*1.1. The resulting phylogenetic hypothesis had a very high support.

It should be mentioned that some strains identified as ‘*P. mandapamensis*’ based on results of the phylogenetic analysis shown in the Figure 1 were previously classified as *P. leiognathi* based on analysis of luminescence genes [18], [34]. The strains are listed in the Table 1.

### Survey of lineage-specific genes

Availability of the genome sequences of representative strains from *P. leiognathi* and ‘*P. mandapamensis*’ allowed searching for sequences specific to either of the two lineages that could have a role in bioluminescent symbioses. This comparative analysis identified ‘*P. mandapamensis*’ *svers*.1.1 or *P. leiognathi lrivu.*4.1-specific genes, while eliminating sequences present in *P. angustum* S14.

Pairwise reciprocal BLASTP of ‘*P. mandapamensis*’ *svers.*1.1, *P. leiognathi lrivu.*4.1 and *P. angustum* S14 (see Material and Methods for the details) found 714 CDSs unique to *P. leiognathi lrivu*.4.1 and 276 CDSs unique to ‘*P. mandapamensis*’ *svers*.1.1. The CDSs unique to the *P. leiognathi lrivu.*4.1 included 73 transposases and 151 out of the 162 CDSs present on the 159 kb plasmid. The 151 unique CDSs found on *P. leiognathi lrivu*.4.1 plasmid included genes of the *lux-rib*_2_ operon. ‘*P. mandapamensis*’ *svers*.1.1 specific CDSs included the *luxF* and *lumP* genes that were found upstream of the luminescence operons of ‘*P. mandapamensis*’, but are absent in the luminescence operons of *P. leiognathi*.

*P. leiognathi lrivu.*4.1-specific CDSs included high percentage of CDSs in the COG category L; specifically, 99 unique CDSs (Table 2). 59% of the *P. leiognathi lrivu.*4.1 specific CDSs and 55% of ‘*P. mandapamensis*’ *svers*.1.1 specific CDSs could not be assigned to any COG category. Otherwise CDSs specific to both strains were functionally broadly distributed, with none of the COG categories overrepresented in either of the strains (Table 2).

**Figure 2:**
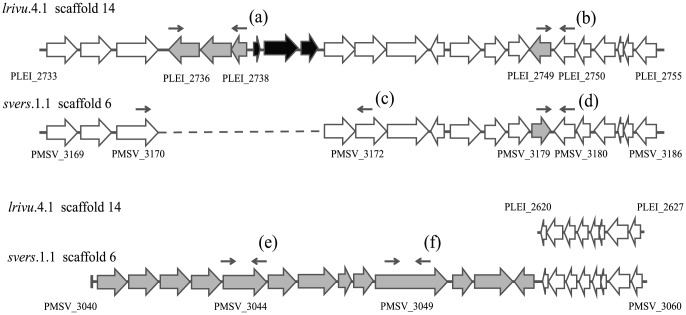
Comparison of exopolysaccharide biosynthesis genes in *P. leiognathi lrivu*.4.1 and ‘*P. mandapamensis*’ *svers.*1.1. Large arrows represent CDSs predicted from the genome annotation, arrows direction indicate the direction of transcription. White arrows indicate orthologs found in both strains. Grey arrows represent strain-specific sequences predicted to take part in exopolysaccharide biosynthesis. Grey arrows on ‘*P. mandapamensis*’ *svers.*1.1 scaffold 6 indicate CDS with homology to genes of *A. fischeri syp* operon. Black arrows mark three CDSs specific to *P. leiognathi lrivu*.4.1 that have no predicted function in exopolysaccharide biosynthesis. Dotted line indicates a region absent in ‘*P. mandapamensis*’ *svers*.1.1, vertical line indicates end of the scaffold sequence. Small arrows indicate primers used in testing the genes incidence in *P. leiognathi* and ‘*P. mandapamensis*’. Letters in brackets correspond to those used in the Table 1.

Among sequences unique to either of the two symbiotic strains we found a high number of CDSs predicted to have a function in exopolysaccharide biosynthesis. These sequences included 15 glycosyltransferases, 7 unique to *P. leiognathi lrivu.*4.1 and 8 unique to ‘*P. mandapamensis*’ *svers.*1.1. Furthermore, strain ‘*P. mandapamensis*’ *svers*.1.1 has a 13-gene locus (gene loci PMSV_3040 to PMSV_3052), which included glycosyltransferases and regulatory sequences with a homology to *syp* (symbiotic proteins) of *Aliivibrio fischeri* [35] (Figure 2). The 13-gene locus was absent in *P. leiognathi lrivu*.4.1, but homologs of the genes were found in *P. angustum* S14.

The diversity of CDSs predicted to have a function in exopolysaccharide biosynthesis, as well as the predicted role of the genes in some *Vibrionaceae*-animal associations [35]–[36] suggested that incidence of the genes could provide a clue about ecological diversification of *P. leiognathi* and ‘*P. mandapamensis*’. In order to analyze incidence of the exopolysaccharide biosynthesis formation genes in the two lineages, selected genes were amplified in 23 additional strains from the two lineages. The diagram of the amplification scheme can be found in Figure 2. The results of amplification are summarized in Table 1. The analysis revealed a high diversity of the exopolysaccharide biosynthesis genes composition in both lineages. Symbionts isolated from the same host species (for example *Nuchequula nuchalis* symbionts) had different exopolysaccharide biosynthesis genes composition and at least in one case, strains isolated from light organ of the same fish specimen (i.e. strains 220710F10A and 220710F10B) had different composition of the genes (Table 1).

PCR reactions designed to amplify sequences homologous to the *A. fischeri syp* operon produced a product in all analyzed ‘*P. mandapamensis*’ strains, except for the strains *ajapo.*4.20, in which PCR reaction designed to amplify a homolog of PMSV_3049 (fragment f in the Figure 2) did not result in any product. In contrast, the same PCR reactions did not produce any amplicons when genomic DNA of 10 *P. leiognathi* strains were used as templates (Table 1). These results suggest that the 13-gene locus with homology to *syp* genes of *A. fischeri* is ‘*P. mandapamensis*’ specific.

## Discussion

This study was initiated to analyze the diversification of two lineages of bioluminescent symbionts, *P. leiognathi* and ‘*P. mandapamensis*’, by using genome-level comparison of representative strains form both lineages. The two lineages are phylogenetically and ecologically closely related, and offer a unique opportunity to analyze early stages of bacterial speciation. *P. leiognathi* and ‘*P. mandapamensis*’ are particularly interesting as a model for research on bacterial speciation because the two lineages apparently diverged only recently and are at the cut-off point used for taxonomic definition of bacterial species. Recent divergence of the two lineages can be deducted from the level of genomic relatedness (predicted from the DDH and ANI), results of phylogenetic analyses (Figure 1), strain-to-strain variation in the exopolysaccharide biosynthesis genes incidence (Table 1), overlapping ecological niche (i.e. symbiotic hosts, see Table 1 and [6]), and higher frequency of exchange of luminescence genes between the two lineages than between other *Photobacterium* species [19].

We are aware that according to strict bacterial taxonomy rules, ‘*P. mandapamensis*’ should be classified as a later, heterotypic synonym of *P. leiognathi*. DNA-DNA relatedness values for strains ATCC 25521^T^ and ATCC 27561^T^ and ANI calculated for genome sequences of ‘*P. mandapamensis*’ *svers*.1.1 and *P. leiognathi lrivu*.4.1 are at the proposed cut-off point for taxonomic description of new bacterial species, and do not allow to validly describe ‘*P. mandapamensis*’ as a species different from *P. leiognathi*. It is also not clear if the two lineages can be described as subspecies, since subspecies designation requires that groups of strains exhibit consistent phenotypic variation within species [37], but no diagnostic phenotypic traits can distinguish *P. leiognathi* strains from ‘*P. mandapamensis*’ [15]–[16]. It is also difficult to apply the ecotype-based species concept [38] to classification of the two lineages, since there are no apparent differences between the ecological niche of *P. leiognathi* and ‘*P. mandapamensis*’, and we could not identify ecologically distinct populations formed by the two lineages. Strains from both lineages can be isolated from the same geographical locations, and they can be found as co-symbionts in light organs of the same fish host (Table 1). However, regardless of taxonomic classification of the bacteria, the current work clearly shows that *P. leiognathi* and ‘*P. mandapamensis*’ form two discrete lineages, which can be diagnosed using molecular phylogeny analysis. These two bacterial lineages do not fit the pragmatic and strict species definition used in bacterial taxonomy. However, we believe that phylogenetic, ecological and genomic studies of such discrete lineages will lead toward more refined bacterial species definition, which will encompass recent advances in bacterial evolutionary theory.

Phylogenetic analysis of orthologs found in *P. leiognathi lrivu*.4.1 and ‘*P. mandapamensis*’ *svers*.1.1 identified two markers that can delineate *P. leiognathi* and ‘*P. mandapamensis*’. Other sequences tested in this study could not separate the analyzed strains into robust, well-supported clades. Lack of phylogenetic resolution when using sequences of some orthologs could result from several factors. Most likely, the two lineages diverged only recently, and orthologs present in both lineages did not have sufficient time to acquire enough mutations to provide sufficient resolution for phylogenetic analysis. Recent diversification of the two lineages can be deducted from a lower genetic distance between core sequences of *P. leiognathi lrivu.*4.1 and ‘*P. mandapamensis*’ *svers.*1.1 compared to genetic distance between core sequences of *P. angustum* and *P. profundum* (see ANI results in Table S3). Also, early stages of *Vibrionaceae* diversification process are predicted to include substantial amount of homologous recombination [39], and finding only limited number of orthologous sequences that distinguish the two lineages could be caused by relatively high rate of homologous recombination between *P. leiognathi* and ‘*P. mandapamensis*’. While the process of diversification will continue, we expect the rate of homologous recombination between both lineages to decrease.

Results of this study allow us to hypothesize about the process of diversification that led to evolution of the two lineages. We found that *P. leiognathi lrivu*.4.1 has 14.6% larger genome than ‘*P. mandapamensis*’ *svers*.1.1. Also, 3,453 CDSs conserved between both strains constitute smaller percentage of total *P. leiognathi lrivu*.4.1 CDSs compared to ‘*P. mandapamensis*’ *svers.*1.1. These results are in contrast to other the analyzed *P. angustum* or *P. profundum* strains, which have nearly identical genome sizes and comparable proportion of CDSs conserved in each species. These findings indicate that *P. leiognathi lrivu*.4.1 has higher genome plasticity and higher rate of foreign gene acquisition compared to ‘*P. mandapamensis*’ *svers.*1.1. If other *P. leiognathi* strains also have highly plastic genomes they could rapidly acquire mutations giving them fitness advantage over ‘*P. mandapamensis*’ in some habitats. This fitness advantage could lead to differential environmental associations between *P. leiognathi* and ‘*P. mandapamensis*’ and eventual diversification of the two lineages, akin to ecological diversification observed in *V. cyclitrophicus* [39]. However, we were unable to identify differences between habitats of the two lineages. Previous studies found some differences in symbiotic host range between the two lineages [6], but we found no evidence for bioluminescent symbioses role in diversification of the two lineages (see below).

It should be mentioned that the rate of gene transfer from *P. leiognathi* to ‘*P. mandapamensis*’ could be different from the rate of gene transfer from ‘*P. mandapamensis*’ to *P. leiognathi*. Some of the strains identified in this work as ‘*P. mandapamensis*’ were previously incorrectly identified as *P. leiognathi*, based on the presence of luminescence genes horizontally transferred from *P. leiognathi* (Table 1) [2], [19]. However, previous studies as well as this work found no evidence for transfer of luminescence genes from ‘*P. mandapamensis*’ to *P. leiognathi*. This transfer of luminescence genes in one direction, i.e. the genes are more frequently transferred from *P. leiognathi* to ‘*P. mandapamensis*’ than vice-versa, suggests that the rate of exchange of luminescence genes might be different between both lineages. If the overall rate of gene transfer between *P. leiognathi* and ‘*P. mandapamensis*’ is comparable to the rate of luminescence genes exchange, i.e. ‘*P. mandapamensis*’ more frequently acquire *P. leiognathi* sequences than vice versa, different rates of gene transfer could lead to divergence of two distinct lineages. However, testing of this hypothesis will require a more detailed analysis of additional whole genome sequences of strains from both lineages and from related species.

In contrast to the predicted role of recombination and horizontal gene transfer during diversification of the two lineages, we were unable to find evidence showing that bioluminescent symbioses with specific host animals played a role in the diversification of *P. leiognathi* and ‘*P. mandapamensis*’. Initial analysis of strain-specific genes suggested a role of exopolysaccharide metabolism genes in *P. leiognathi* and ‘*P. mandapamensis*’ associations with specific host animals, similar to the genes function during *Aliivibrio fischeri* associations with fish and squids [36]. However, survey of the genes diversity in 25 strains from both lineages found no apparent correlation between presence or absence of a specific exoplysaccharide biosynthesis genes and the bacterial ability to colonize light organs of a specific fish host. This result suggests a different mechanism of host animal colonization than that predicted for *Aliivibrio fischeri*, in which the presence or absence of specific exopolysaccharide biosynthesis genes and their regulators affects *A. fischeri* ability to colonize specific host animals [36].

## Supporting Information

Figure S1 **Identification of potential markers for phylogenetic analysis of** ***P. leiognathi*** **and ‘** ***P. mandapamensis*** **’.**
(EPS)

Figure S2 **Phylogenetic analyses using maximum-likelihood and neighbor-joining algorithms.** Analyses of the concatenated PMSV_2285 and PMSV_ 4043 alignment were done in PAUP* version 4.0b10 for UNIX. Neighbor-joining analysis (left) used UPGMA algorithm. Maximum-likelihood analysis (right) used 100 replicates of heuristic search with the likelihood criterion. Jackknife resampling values after 1000 (neighbor-joining) or 100 (maximum-likelihood) replicates are shown at the nodes, some omitted for clarity.
(EPS)

Table S1 **Primer sequences for amplification of genetic sequences used in this study.** ‘*P. mandapamensis*’ *svers*.1.1 genes have the locus tag PMSV and *P. leiognathi lrivu*.4.1 genes have the locus tag PLEI.
(DOCX)

Table S2 **Accession numbers for sequences used in the study.** For strains with whole genome sequences available the locus tag is provided instead.
(DOCX)

Table S3 Average nucleotide identities.
(DOCX)
