# Supplementary material for: Diversification of Two Lineages of Symbiotic Photobacterium
Source: PLoS One. 2013 Dec 13;8(12):e82917. doi: 10.1371/journal.pone.0082917 (PMC3862722; doi:10.1371/journal.pone.0082917)
Supplement: Table S1 — Primer sequences for amplification of genetic sequences used in this study. ‘P. mandapamensis’ svers.1.1 genes have the locus tag PMSV and P. leiognathi lrivu.4.1 genes have the locus tag PLEI. (DOCX) [file pone.0082917.s003.docx]

Supporting Table S1

Primer sequences for amplification of genetic sequences used in this study. ‘*P. mandapamensis*’ *svers*.1.1 genes have the locus tag PMSV and *P. leiognathi* *lrivu*.4.1 genes have the locus tag PLEI.

| Gene(s) | Primer name | Primer sequence 5'-3' | Note |
| --- | --- | --- | --- |
| Primers used for amplification of sequences used in phylogenetic analyses shown in the Figure 1 and in the Supporting Figure S1. | | | |
| PMSV_2285/PLEI_3531 | PMSV_2285fw | ACCGCCAGCATTTGATGAAG |  |
|  | PMSV_2285rv | CACTGATGGTTTCGAGTGTTCC |  |
| PMSV_4043/PLEI_1315 | PMSV_4043fw | GGTGTTTGCCATGTTGAGTC |  |
|  | PMSV_4043rv | TCGTTTGTCTCACCATGTTCAG |  |
| PMSV_564/PLEI_1316 | PMSV564f | CTATGGCTGGTATATGGGTAAC |  |
|  | PMSV564r | CCAATAAATTCAGCATCTTGCTC |  |
| PMSV_566/PLEI_1844 | PMSV556f | GAGTTCGTTATTGGAAGTCG |  |
|  | PMSV556r | ACCACTTCGCCATCTTGCATC |  |
| PMSV_1350/PLEI_0794 | PMSV1350f | CGGAGAACAAGAAAAGCGAT |  |
|  | PMSV1350r | CATCCTTCTACCGCACCAGCAA |  |
| PMSV_3467/PLEI_2546 | PMSV3467f | GCAATGCGCCAGTAAGTAAAGG |  |
|  | PMSV3467r | CGTGTTGTCATCTAAGAATTG |  |
| Primer sequences used for amplification of the exopolysaccharide biosynthesis genes. Amplification scheme is shown in the Figure 2. | | | |
| PLEI_2736 to PLEI_2738 | PLEI_2736fw | TGATGGCTATCCAGACTATCC | Fragment a in the Figure 2 |
|  | PLEI_2738rv | TCGTTTGTCTCACCATGTTCAG |  |
| PLEI_2749 to PLEI_2750 | PLEI_2749fw | GACTCAATACCACCATGATGC | Fragment b in the Figure 2 |
|  | PLEI_2750rv | ATCGTTGAAGGTGTAATGCG |  |
| PMSV_3170 to PMSV_3172 | PMSV_3170fw | GAGCAATACGTTGGTGAACGTC | Fragment c in the Figure 2 |
|  | PMSV_3172rv | CTTGGGTGATAATAGTGACTTCG |  |
| PMSV_3179 to PMSV_3180 | PMSV_3179fw | ACCGATAGTGACTGTATTCC | Fragment d in the Figure 2 |
|  | PMSV_3180rv | ATCGTTGAAGGTGTAATGCG |  |
| PMSV_3044 | PMSV_3044fw | GTACAGATGAGTTTGACTTGG | Fragment e in the Figure 2 |
|  | PMSV_3044rv | GATTGATGGCATCGATTTCAG |  |
| PMSV_3049 | PMSV_3049fw | GCTATAAGCCTGAAATGAATGTGG | Fragment f in the Figure 2 |
|  | PMSV_3049rv | GCGGATACTCGATATATTGGC |  |
