# Supplementary material for: Diversification of Two Lineages of Symbiotic Photobacterium
Source: PLoS One. 2013 Dec 13;8(12):e82917. doi: 10.1371/journal.pone.0082917 (PMC3862722; doi:10.1371/journal.pone.0082917)
Supplement: Table S2 — Accession numbers for sequences used in the study. For strains with whole genome sequences available the locus tag is provided instead. (DOCX) [file pone.0082917.s004.docx]

Supporting Table S2.

Accession numbers for sequences used in the study. For strains with whole genome sequences available the locus tag is provided instead.

| Strain | PMSV_2285 homolog accession number | PMSV_4043 accession number |
| --- | --- | --- |
| *P. leiognathi lrivu*.4.1 | PLEI_3531 | PLEI_1315 |
| ‘*P. mandapamensis*’ *lrivu*.3.1 | JX893638 | JX893615 |
| ’*P. mandapamensis*’ 220710F8A | JX893639 | JX893616 |
| ’*P. mandapamensis*’ 220710F9A | JX893640 | JX893617 |
| ’*P. mandapamensis*’ 220710F10A | JX893641 | JX893618 |
| ’*P. mandapamensis*’ 220710F10B | JX893642 | JX893619 |
| *P. leiognathi lnuch*.13.1 | JX893643 | JX893620 |
| *P. leiognathi lnuch*.21.1 | JX893644 | JX893621 |
| *P. leiognathi* 170910FA1 | JX893645 | JX893622 |
| ’*P. mandapamensis*’ 170910FB1 | JX893646 | JX893623 |
| *P. leiognathi* 170910FC1 | JX893647 | JX893624 |
| ’*P. mandapamensis*’ *svers*.1.1 | PMSV_2285 | PMSV_4043 |
| ’*P. mandapamensis*’ *svers*.9.9 | JX893648 | JX893625 |
| *P. leiognathi* 220710F2A | JX893649 | JX893626 |
| ’*P. mandapamensis*’ 220710F3A | JX893650 | JX893627 |
| *P. leiognathi* 220710F4A | JX893651 | JX893628 |
| *P. leiognathi* 220710F5A | JX893652 | JX893629 |
| ’*P. mandapamensis*’ *ajapo*.3.1 | JX893653 | JX893630 |
| ’*P. mandapamensis*’ *ajapo*.4.20 | not amplified | JX893631 |
| *P. leiognathi* ATCC 25521T | JX893654 | JX893632 |
| ’*P. mandapamensis*’ *ppana*.3.1 | JX893655 | JX893633 |
| *P. leiognathi lelon*.2.1 | JX893656 | JX893634 |
| ’*P. mandapamensis*’ PL-721 | JX893657 | JX893635 |
| ’*P. mandapamensis*’ NCCB 80036 | JX893658 | JX893636 |
| ’*P. mandapamensis*’ ATCC 27561T | JX893659 | JX893637 |
| *P. angustum* S14 | VAS14_13814 | VAS14_23079 |
| *P. angustum* SKA34 | SKA34_10068 | SKA34_00740 |
| *P. damselae* CIP 102761 | VDA_000880 | VDA_003117 |
| *P. profundum* SS9 | PBPRB0584 | PBPRA3475 |
| *P. profundum* 3TCK | P3TCK_06367 | P3TCK_23613 |
| *A. fischeri* ES114 | VF_A1168 | VF_2468 |
| *A. fischeri* MJ11 | VFMJ11_A1262 | VFMJ11_2591 |
| *V. orientalis* CIP 102891T | VIA_001574 | VIA_000584 |
| *V. splendidus* ATCC 33789 | VISP3789_21528 | VISP3789_20223 |
