# Supplementary material for: Diversification of Two Lineages of Symbiotic Photobacterium
Source: PLoS One. 2013 Dec 13;8(12):e82917. doi: 10.1371/journal.pone.0082917 (PMC3862722; doi:10.1371/journal.pone.0082917)
Supplement: Table S3 — Average nucleotide identities. (DOCX) [file pone.0082917.s005.docx]

Supporting Table S3. Average nucleotide identities.

|  |  | 1 | 2 | 3 | 4 | 5 | 6 | 7 | Sequence accession number |
| --- | --- | --- | --- | --- | --- | --- | --- | --- | --- |
| 1 | '*Photobacterium mandapamensis' svers.*1.1 | --- | **96.8** | 81.08 | 81.47 | 73.02 | 72.82 | 73.25 | BACE01000001- BACE01000031 |
| 2 | *P. leiognathi lrivu*.4.1 | **96.6** | --- | 82.18 | 82.5 | 73.15 | 73.00 | 73.66 | BANQ01000001 - BANQ01000184 |
| 3 | *P. angustum* SKA34 | 81.04 | 82.44 | --- | **93.41** | 72.88 | 72.75 | 73.04 | AAOU01000001 - AAOU01000088 |
| 4 | *P. angustum* S14 | 81.44 | 82.54 | **93.41** | --- | 73.03 | 72.85 | 73.5 | AAOJ01000001 - AAOJ01000045 |
| 5 | *P. profundum* SS9 | 73.14 | 74 | 73.24 | 73.36 | --- | **92.89** | 72.85 | NC_006370.1, NC_006371.1, NC_005871.1 |
| 6 | *P. profundum* 3TCK | 72.59 | 73.94 | 72.67 | 72.76 | **92.73** | --- | 72.32 | AAPH01000001- AAPH01000082 |
| 7 | *P. damselae* subsp*. damselae* CIP 102761^T^ | 73.36 | 74.46 | 73.11 | 73.6 | 72.63 | 72.65 | --- | ADBS01000001- ADBS01000008 |

ANI values are presented as percentage, ANI for strains classified as members of the same species are in bold.
